# Supplementing Environmental Assessments with Cumulative Effects Scenario Modeling for Grizzly Bear Connectivity in the Bow Valley, Alberta, Canada

**DOI:** 10.1007/s00267-022-01720-w

**Published:** 2022-09-30

**Authors:** Matt Carlson, Hilary Young, Adam Linnard, Max Ryan

**Affiliations:** 1 Integral Ecology Group, Ottawa, Ontario Canada; 2 Yellowstone to Yukon Conservation Initiative, Canmore, Alberta Canada; 3 Integral Ecology Group, Duncan, British Columbia Canada

**Keywords:** Grizzly bear, Cumulative effects, Environmental assessment, Scenario modeling, Alberta

## Abstract

Persistence of sensitive wildlife in populated regions requires conservation strategies that address gradual expansion of development footprint and human activity. The project-based environmental assessment regime for municipal development is poorly suited to provide necessary strategic perspective, given its focus on local and short-term impacts. We used the ALCES cumulative effects model to strategically assess impacts to grizzly bears (*Ursus arctos*) in the Bow Valley of Alberta, Canada. Landscape simulation mapped expansion of past and potential future development footprint in the region over multiple decades. Consequences to movement connectivity for grizzly bears were estimated by applying a least cost path analysis to the landscape simulation. An index of recreational activity was derived from fitness tracking data and integrated with the landscape simulation to model change in recreational activity through time. Maps of grizzly bear connectivity and recreational activity were combined to calculate human-bear conflict risk. The analysis suggests that connectivity has been altered through displacement to upslope areas by settlement expansion, such that surrounding natural areas have become important for grizzly bear connectivity. These areas are also popular for outdoor recreation, resulting in elevated human-bear conflict risk which can be expected to increase if development and human activity continue to expand in high connectivity areas. Conservation of wildlife in populated regions will be supported by broadening the scope of environmental assessment to address cumulative effects of development footprint and human activity over large spatial and temporal scales.

## Introduction

Scientists have long recognized that to protect biodiversity, including populations of wide-ranging mammals, large-landscape habitat connectivity must be prioritized (Preston 1962a; Preston 1962b; MacArthur and Wilson 1967). Grizzly bears (*Ursus arctos*) in western Canada, for example, have large home ranges (averaging 1800 km^2^ for males and 700 km^2^ for females; COSEWIC 2012) and subadult male grizzly bears may travel an area as large as 2505 km^2^ to access food and mating opportunities (Graham and Stenhouse 2014). When grizzly bear habitat becomes isolated or fragmented by roads or development, grizzly bear populations risk experiencing long-term stress (Bourbonnais et al. 2013) and genetic isolation (Gibeau 2000; Northrup et al. 2012; Proctor et al. 2012; Sawaya et al. 2013) and may risk extirpation.

In Alberta, grizzly bears have been provincially designated as threatened since 2010. The *Alberta Grizzly Bear Recovery Plan* suggests that recovery depends on permanently secure habitat, habitat connectivity, and reduced anthropogenic mortality throughout grizzly bear range in the province (Alberta Environment and Parks 2020). However, project-based environmental assessments made by municipalities, counties, and municipal districts in Alberta typically only consider potential project impacts at the local scale over the short term. In general, project-level environmental assessments follow Terms of Reference (TORs) agreed upon by the project proponent and the decision-making body responsible for project approval. TORs for an environmental assessment outline the indicators against which environmental impacts are to be measured, and establish the geographic scope of relevance to the decision-making body. At most, the scope of the assessment may extend to the jurisdictional boundaries of that body’s authority. More often, the scope is limited to the lands proposed for development and the area immediately adjacent to them. For small municipalities with limited budgets, a decision to set TORs for environmental assessment with narrow geographic scope may be economically-driven, while limited capacity may prevent emergent environmental concerns or conditions from being evaluated if not already identified in the TOR. The consequence of limited TORs is that the studies that result seldom reflect a project’s full ecological context, including the direct (e.g., habitat loss) and indirect effects (e.g., habitat alienation, human-wildlife conflict) of the project to key ecological attributes, as well as the effects of other existing and foreseeable projects within the region (Noble et al. 2017; Dibo et al. 2018).

Borrowing from economist Alfred E. Kahn, environmental cumulative effects are often described as the “tyranny of small decisions” (Odum 1982): the environmental change caused by many activities across time and space (Canadian Council of Ministers of the Environment 2014). One challenge of cumulative effects is that degradation can accumulate on a larger scale than is evaluated by environmental assessments. Consequently, project-based environmental assessments may adequately address the questions outlined in their TOR, but inadequately address the broader objective of maintaining the ecological health of the region; this can be true even of assessments that include a Cumulative Effects Assessment (CEA) (Dubé 2013). Large-landscape habitat connectivity is a particular challenge for environmental assessments because wildlife and ecological systems operate beyond the scope of individual projects and the regulatory boundaries of a jurisdiction. Alberta does not currently require the cumulative effects at a regional scale to be assessed as part of project EA requirements. Therefore, such analyses are left to conservation groups or other institutions existing outside of the formal regulatory process.

Simulation modeling is a tool meant to help address this incompatibility of scope, and to measure the difficult-to-measure gains of “avoided ecological loss” (Carlson et al. 2019). By illustrating the potential ramifications of multiple changes on key environmental indicators, cumulative effects scenario modeling can show the comparative impact of different potential decisions as well as the cumulative impact of multiple decisions. More specifically, if regional land managers can jointly identify the environmental future they want to maintain or achieve (e.g., grizzly bears can move safely through a region), simulation modeling can shed light on the costs and benefits associated with different means of achieving that conservation goal. In addition to community engagement processes and thorough and meaningful Indigenous consultation, cumulative effects scenario modeling can help decision-makers make well-informed decisions that prioritize long-term environmental outcomes.

The Bow Valley, in Treaty 7 territory of Alberta, Canada, is a wide, productive, low-elevation valley that provides habitat and acts as a movement corridor for ungulates and carnivores (Minister of Supply and Services 1996; Bow Valley Human-Wildlife Coexistence Roundtable 2018). The Bow Valley is also a key east-west connector located close to the center of the Yellowstone to Yukon (Y2Y) region, which runs 3400 km along the Rocky Mountains from Yellowstone National Park in Wyoming, United States, to the Arctic Circle in Yukon, Canada, and is one of the last remaining intact mountain ecosystems in the world. However, valleys with gravel-bed river floodplains, like the Bow Valley, have been disproportionately affected by human infrastructure and activities (Hauer et al. 2016). Along with Banff National Park and a number of provincial protected areas, the Bow Valley houses two major highways, a railway, two towns, three hamlets, and a growing residential, industrial and recreational footprint. The Town of Canmore’s permanent population has grown from 3166 in 1980 to 15,990 in 2021 and existing development proposals could again double the town’s population and significantly increase its development footprint. Recent modeling indicates that human development has already reduced the connectivity value of the Bow Valley by an average of 85% from the historical state for grizzly bears and grey wolves (*Canis lupus*) (Whittington et al. 2022).

Recent development proposals in the Bow Valley include a sightseeing gondola from Canmore with casino, resort and conference center (Colgan 2022); a residential, resort, and commercial development that would nearly double Canmore’s population (Anderson 2022); a passenger rail service between Calgary Airport and Banff (Cryderman 2022); and a sightseeing gondola from Banff to an existing ski resort (Ellis 2021). Each of these has or will require some level of project-based EA, whether at the municipal, provincial or federal level. Still, none of these have triggered a CEA within formal planning processes, and thus far none have been assessed for cumulative effects at a regional scale.

To better understand the impact of human development and recreation on grizzly bear movement paths and the risk of human-bear conflict in the Bow Valley, we modeled regional cumulative effects for the past, present, and foreseeable future as an important supplement to the limited scope of standard small-scale environmental assessments. Additionally, we considered how three generalized land management scenarios could change the trajectory of the model’s results. Ultimately, this paper considers the promise and limitations of cumulative effects scenario modeling in better including and informing multi-jurisdictional decision-makers, with a goal of better meeting the needs of wide-ranging wildlife species.

## Methods

The cumulative effects of land use to grizzly bear connectivity were assessed by: a) modeling past (1970s to current) and potential future (current to 2050s) changes in land-use footprints and recreational activity; b) applying least cost path analysis to estimate the effect of footprint growth on connectivity; and c) integrating least cost paths and estimates of recreational activity to map spatial and temporal changes in the risk of human-bear conflict. The analysis was completed using ALCES Online, a decision-support tool that supports cumulative effects assessment through web-based delivery of spatial landscape simulation (Carlson et al. 2014). ALCES Online’s landscape simulator exposes a cell-based representation of space to scenarios that differ with respect to the rate and spatial pattern of development. A raster calculator is used to apply methods to simulated landscape dynamics to calculate and map the performance of metrics such as wildlife habitat through space and time. The tool’s landscape simulator and indicator calculator are designed to provide the user with a high level of control when defining scenarios and indicator models. This flexibility has seen the tool used in a range of geographies and contexts such as urban planning, land-use planning, regional environmental assessment, wildlife management, and conservation planning (Rempel et al. 2021, Carlson et al. 2019, Leston et al. 2020, Carlson and Stelfox 2014, Carlson et al. 2015).

**Fig. 1:**
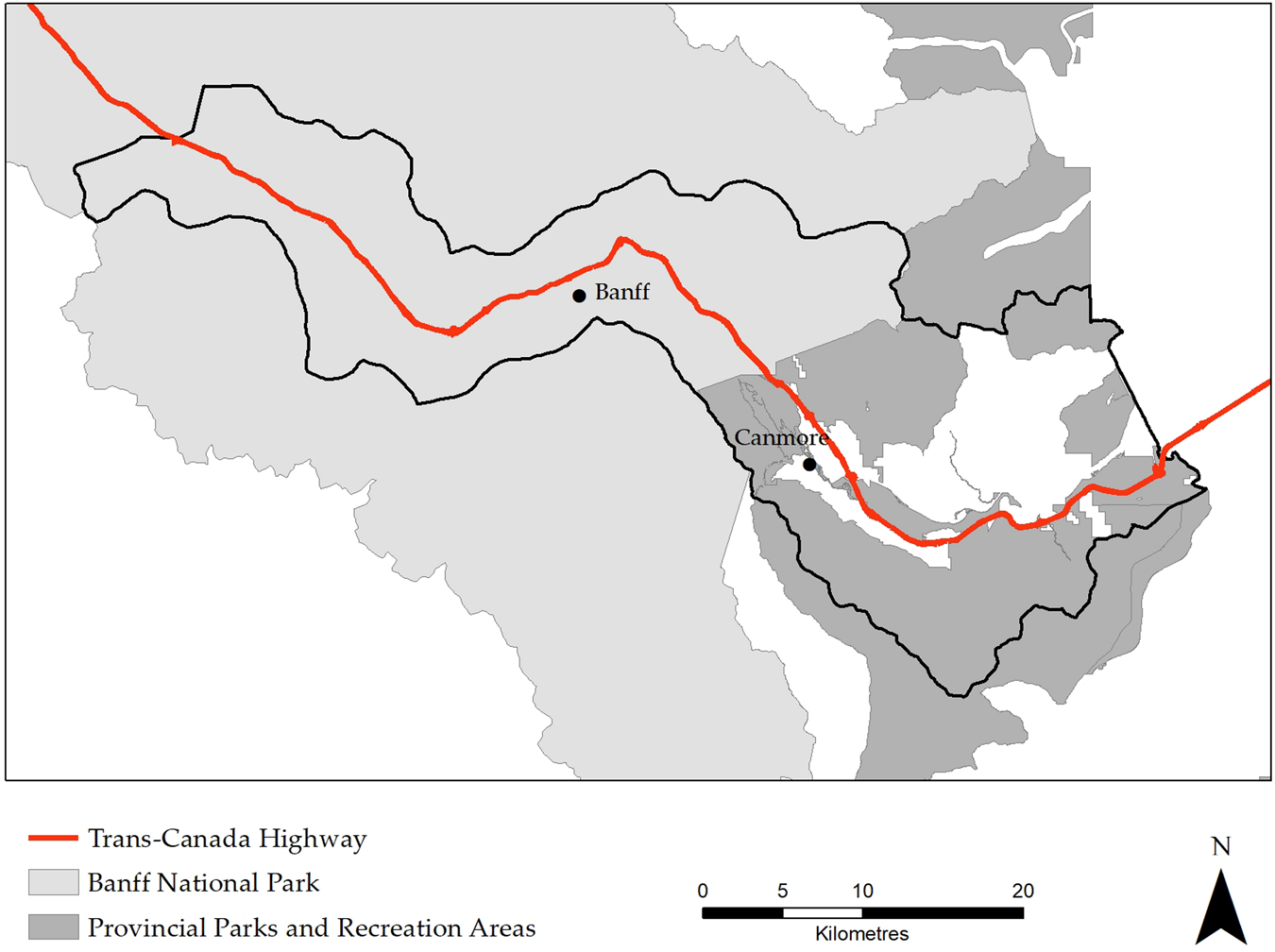
The study area boundary relative to the location of the towns of Canmore and Banff, the Trans-Canada Highway, and protected areas

The scenario modeling was completed for a 900 km^2^ portion of the Bow Valley extending from Castle Junction to the Kananaskis River that includes the towns of Banff and Canmore, the Trans- Canada Highway, and multiple protected areas (Fig. 1). The study area is the same as that used during a previous assessment of human-wildlife conflict risk in the region (Bow Valley Human-Wildlife Coexistence Roundtable 2018). Current landscape composition was estimated by integrating inventories of natural and anthropogenic land cover (Alberta Biodiversity Monitoring Institute and Alberta Human Footprint Monitoring Program 2020, Centre for Topographic Information 2009), as well as recreational trail network data provided by Parks Canada and Alberta Environment and Parks that included designated as well as informal (non-legal) trails. The resulting dataset represented landscape composition as the proportion of each 100 m cell that is covered by each natural and anthropogenic cover type. We estimated historical landscape composition at four intervals (1970, 1990, 2000, 2010) by removing footprints from the current landscape composition layer based on date of origin information. Sources for footprint date of origin included Agriculture and Agri-Food Canada Land Use maps for 1990, 2000, and 2010 (Agriculture and Agri-Foods Canada 2015), the Canada Land Inventory (Agriculture and Agri-Food Canada 1998), the ABMI Human Footprint Inventory (Alberta Biodiversity Monitoring Institute and Alberta Human Footprint Monitoring Program 2020), and Wikipedia pages for major recreational developments.

Simulation of future landscape dynamics over the next three decades focused on settlement expansion. Rural settlement footprint grew by 61% over 30 years based on the projected population growth rate of the Municipal District (MD) of Bighorn (Urban Futures 2017). The footprint was simulated to grow outwards from existing footprint with a higher likelihood of new development in proximity to larger patches of existing footprint. Settlement footprint in the town of Canmore grew by 87% over 30 years, which is 10% lower than the town’s projection population growth rate of 97% (Urban Futures 2017) to reflect the objective of accommodating about 10% of population growth within the town’s existing footprint (Town of Canmore 1998). Simulated expansion of Canmore occurred outwards from the town center, initially within the Growth Boundary and then within two areas that have been proposed for future town expansion referred to as the Area to Be Determined in planning documents (Town of Canmore 2016). The Town of Banff’s settlement footprint remained fixed during the forecast because expansion is not allowed due to the town’s location within Banff National Park.

We estimated current recreational activity using the Strava Global Heatmap (Strava 2021), which represents the intensity of public activities recorded on GPS-enabled devices by Strava users. We inspected map colors to assign 6 activity levels: 0 (no activity) through 5 (highest level of activity). Recreational use increased exponentially across the activity levels based on comparison of the activity levels with trail use data that was available for a subset of trails in Banff National Park. During simulations, we applied the nonlinear relationship between activity levels and absolute activity such that recreational activity categories changed at a slower rate than simulated changes in absolute recreational activity. Historical recreational activity layers were not available and were instead modeled by applying two rules. First, recreational activity currently within recreational facilities or within 2 km of settlement footprint was assumed to not have existed prior to the developments. According to the Strava data, over 80% of recreational activity is currently located within 2 km of settlement footprint, and the first rule assumes that activity is generated by the nearby development. Second, the intensity of recreational activity elsewhere was assumed to have increased historically such that the overall growth rate of recreational activity in the region matched the historical population growth rate. Projected future growth in recreational activity also matched the population growth rate in Canmore and the MD of Bighorn. Half of the simulated new recreational activity in these municipalities occurred through intensified use of existing trails. The other half of new recreational activity occurred as new trails located within 2 km of new settlement footprint. In Banff, where settlement expansion did not occur during the simulation, intensity of use at existing trails was simulated to grow by 25% per decade, which is conservative compared to the 4.1% per year rate of growth in summer visitation that occurred between 2013 and 2019 (Parks Canada 2021).

In addition to assessing the consequences of a Base Case (i.e., business-as-usual) forecast scenario, we simulated three scenarios to explore sensitivity of outcomes to three general strategies for mitigating human-bear conflict risk: 1) limiting footprint growth; 2) eliminating informal (i.e., non-legal) trail use; and 3) reducing use of trails located away from settlement footprint. The first scenario, referred to as Limited Urban Expansion, excluded future development from undeveloped lands to the east of Canmore’s settlement footprint. Development of these areas accounted for 85% of the expansion of Canmore’s settlement footprint during the Base Case forecast. Human population and recreational activity growth in Canmore and the adjacent Nordic Centre was therefore limited to 15% of the Base Case scenario. The second scenario, referred to as No Informal Trails, eliminated recreational activity from areas that do not overlap with designated trails or with development footprint (settlements, roads, recreation facilities). The third scenario, referred to as Restricted Recreation, applied a 50% reduction to recreational activity (as compared to the Base Case scenario) in areas at least 100 m away from settlement footprint.

We applied least cost path modeling to assess the consequences of past and projected future changes in landscape composition to connectivity patterns. In least cost path modeling, the cost of moving between two points is assessed as the cumulative cost while traversing a raster cost surface (Etherington 2016). Connectivity is assessed to be higher along routes that minimize the cumulative cost of moving between the points. The cost surface was calculated as 1 minus a permeability index that responded to landscape features based on grizzly bear research from the region. A grizzly bear resource selection function (RSF) (Whittington et al. 2021) was applied to past, current, and potential future landscape composition layers and transformed from the log scale to create a 0 to 1 permeability index. A summer, as opposed to winter RSF, was used to focus on habitat preferences when grizzly bear activity is highest. We made two modifications to incorporate the effect of highways on grizzly bear movement. First, the permeability index was multiplied by a factor declining linearly from 1 to 0 as proximity to highways declined from 500 to 0 m based on research that found that grizzly bears avoid roads traveled by more than 100 vehicles per day (Northrup et al. 2012). Second, the Trans-Canada Highway acted as a barrier to least cost paths except at wildlife crossings, the presence of which changed during the simulation as dictated by past and planned future construction dates. The Trans-Canada Highway’s role as a barrier is supported by research from the region (Gibeau 2000) and also reflects that much of the highway is fenced within the study area. The cost surface used the highest resolution supported by input data (100 m) because smaller grain size typically produces better results (Etherington 2016).

To map connectivity between areas utilized by grizzly bears, we generated least cost paths between all pairwise combinations of 100 start points and 100 endpoints randomly selected from cells with at least two grizzly bear locations according to a grizzly bear collar data set (Alberta Environment and Parks 2013). Habitat selection is unlikely to be truly optimal, which we represented by having a cell’s cost equal to a random number selected from a normal distribution with mean equal to its calculated cost and standard deviation equal to the standard deviation in cost across cells in the study area. Ten iterations of each of 10,000 pairwise combinations resulted in 100,000 least cost paths for a landscape, which were summarized as the proportion of the 100,000 paths crossing each cell. The distribution of least cost path proportions was highly skewed with a small number of cells having high values relative to other cells. For consistency with the activity index and to avoid a small number of cells dominating outcomes, the least cost path proportions were divided into six categories with proportion increasing nonlinearly between bins according to the exponential function. These binned proportions are referred to as the connectivity index.

We calculated a human-bear conflict risk index as the product of the connectivity index and the recreational activity level, based on the rationale that overlap between areas of high grizzly bear connectivity and human activity creates risk of human-bear conflict. The connectivity index and recreational activity categories were calculated as 400 m moving-window averages prior to being multiplied together, based on previous research from the region, that applied a 400 m buffer when assessing risk to grizzly bears from non-motorized human activity (Gibeau 1998). A human-bear conflict risk index value greater than 9 was interpreted as high because it suggests a situation where, on average, recreational activity and connectivity exceed moderate values (i.e., 3). A human-bear conflict risk index value greater than 4 but less than or equal to 9 was interpreted as moderate because it suggests a situation where, on average, recreational activity and connectivity exceed low values (i.e., 2). A human-bear conflict risk index value greater than 1 but less than or equal to 4 was interpreted as low because it suggests a situation where, on average, recreational activity and connectivity exceed very low values (i.e., 1). A human-bear conflict risk index value greater than 0 but less than or equal to 1 was interpreted as very low.

The project was conducted in consultation with an advisory group made up of representatives from the Town of Canmore, the Town of Banff, and Alberta Parks to ensure that data was up-to-date, foreseeable future development plans were captured, and that the product was relevant to local and provincial land managers.

## Results

**Fig. 2:**
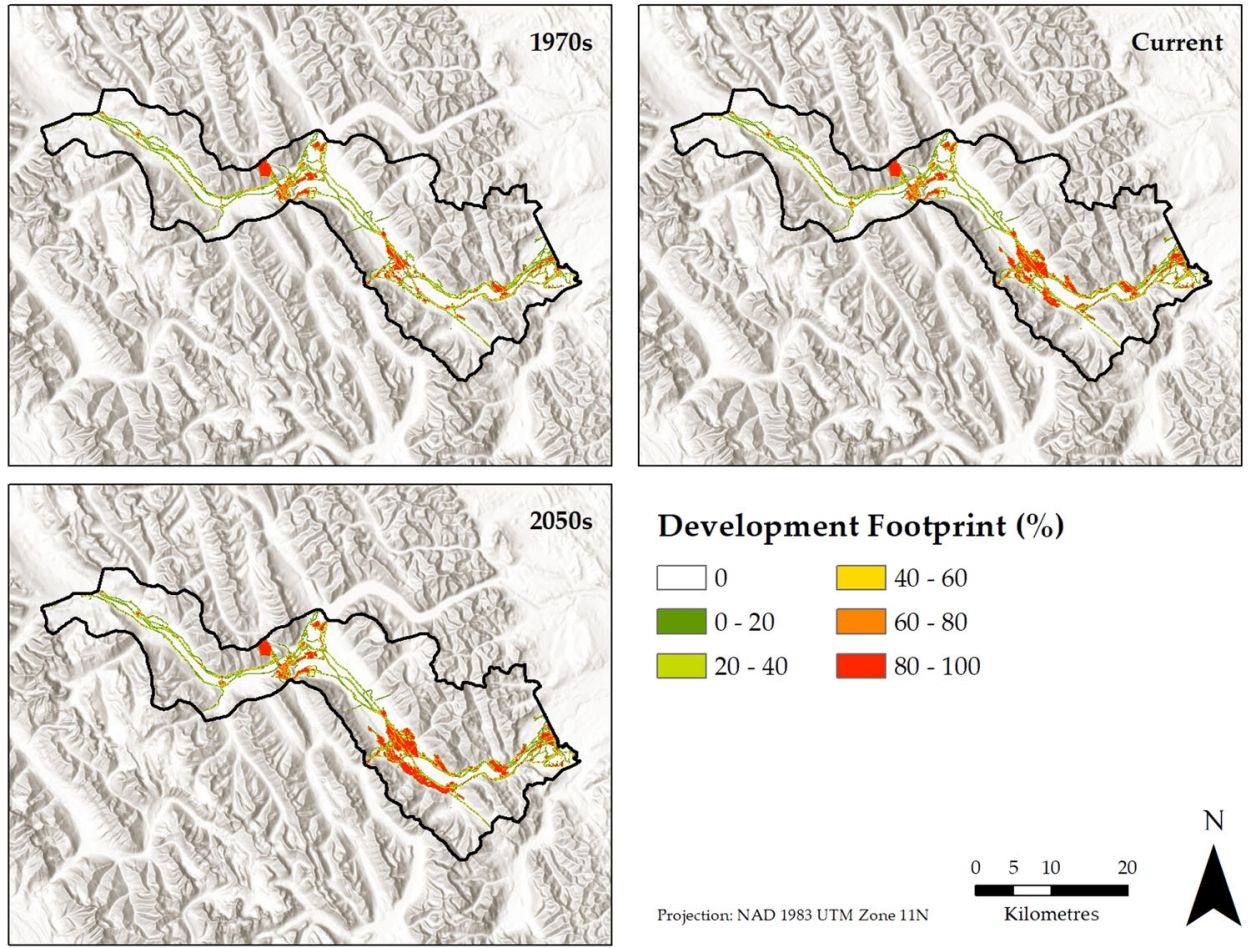
The location of development footprint in the 1970s, today, and the 2050s as projected by a landscape simulation. Development footprint has expanded over the past five decades especially in the vicinity of Canmore, a pattern that is projected to continue in coming decades

Just over five percent of the study area is currently covered by a development footprint, including linear disturbances such as roads, railway, and transmission corridors (18.8 km^2^), recreation facilities such as golf courses and ski areas (12.8 km^2^), settlements (8.4 km^2^), industrial sites such as quarries (7.1 km^2^), and farmland (0.4 km^2^). Footprint increased from 35.1 km^2^ to 47.5 km^2^ over the past five decades and was projected to reach 52.0 km^2^ over the next three decades, with much of the historic and future growth occurring at Canmore (Fig. 2). According to the Strava data, the average recreational activity level (0 to 5 index) in the study area is 0.41. The low value is because much of the study area has a value of 0. The average recreational activity level was simulated to approximately double during the historical period (0.19–0.41) and increase by 34% in the forecast (from 0.41 to 0.55). Recreational activity was concentrated in proximity to Banff and Canmore, with 46% and 48% of current and simulated future (year 2050) recreational activity, respectively, occurring within 2 km of Banff and Canmore settlement footprint. Following the pattern of development footprint growth, simulated growth in recreational activity was most prevalent near Canmore.

**Fig. 3:**
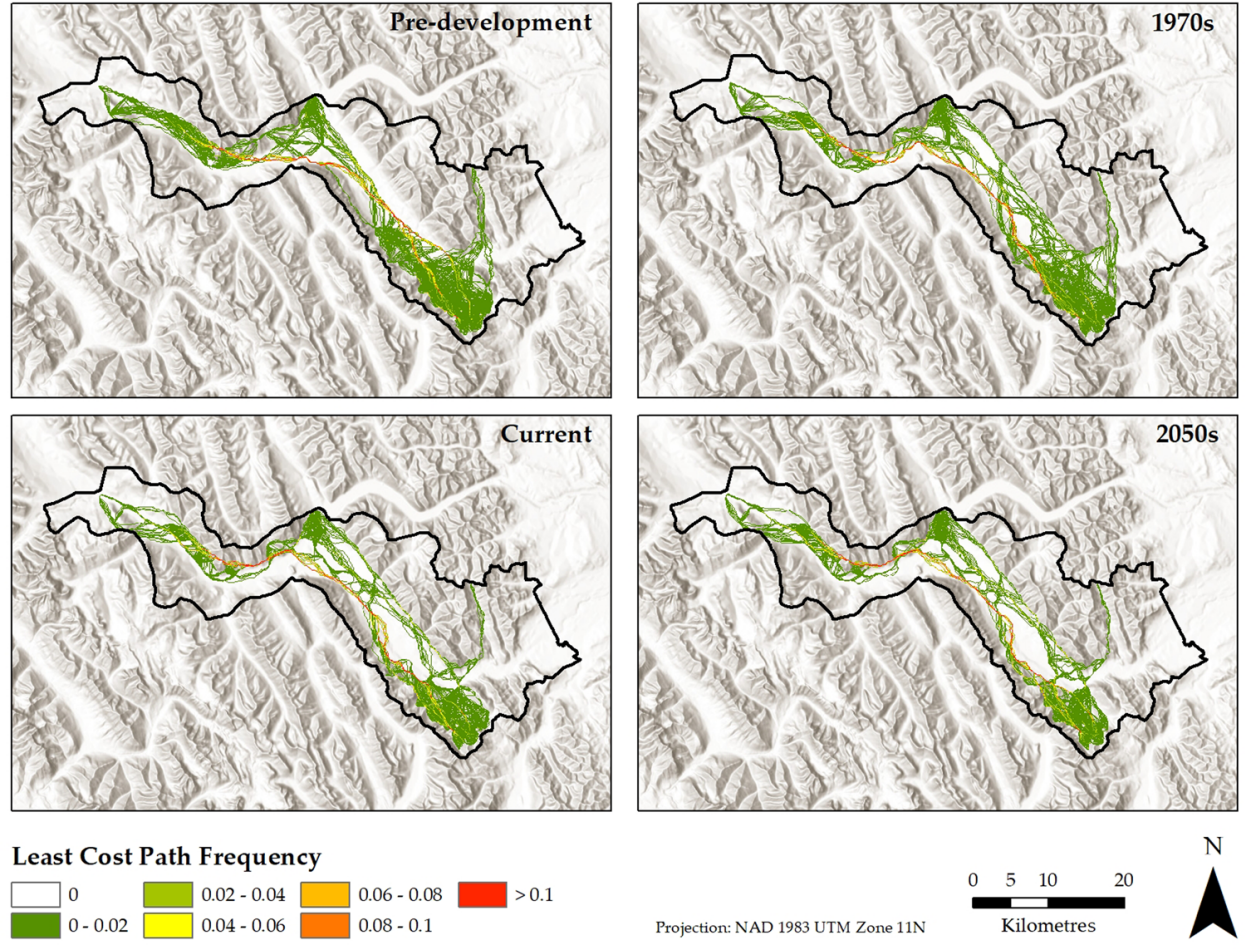
The location of grizzly bear least cost paths as modeled for the pre-development, 1970, current, and 2050 landscapes. The accumulation of development footprint through time has pushed least cost paths outwards from the central portion of the valley

Despite covering a relatively small portion of the study area, development footprint influenced the spatial pattern of least cost paths due to being concentrated along the valley bottom. When development footprint was removed to approximate a pre-development landscape, least cost paths tended to follow the valley bottom, moving upslope only as needed to reach start and end points (Fig. 3). The concentration of least cost paths in the valley was due to higher permeability resulting from lower elevation, flatter topography, and the presence of preferred land cover types as identified by the resource selection function. When development footprint was incorporated, least cost paths were displaced upslope by anthropogenic features such as the Banff and Canmore townsites (Fig. 3). Displacement of least cost paths increased over time as footprint expanded, especially at Canmore where the pace of development was highest. Least cost paths were also sensitive to the Trans-Canada Highway due to its function as a barrier from the 1990s onwards except at wildlife crossings. In response to the barrier, the majority of least cost paths tended to travel on the same side of the highway in between wildlife crossings, thereby isolating habitat located on the opposite side (Fig. 3).

**Fig. 4:**
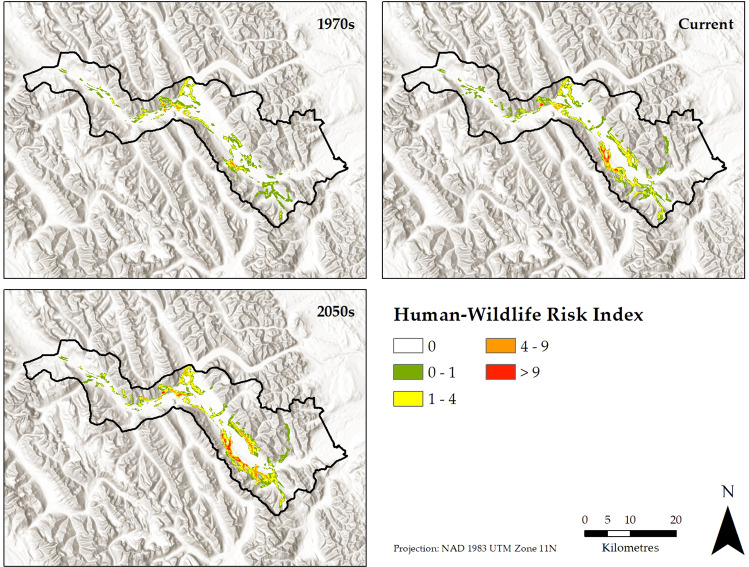
Spatiotemporal changes in the human-bear conflict risk index from the 1970s to the 2050s as calculated from simulations of past and future grizzly bear connectivity and human recreation activity. Higher values of human-bear conflict risk indicate overlap in grizzly bear connectivity and recreation activity, a situation that is assessed to be increasing through time

**Fig. 5:**
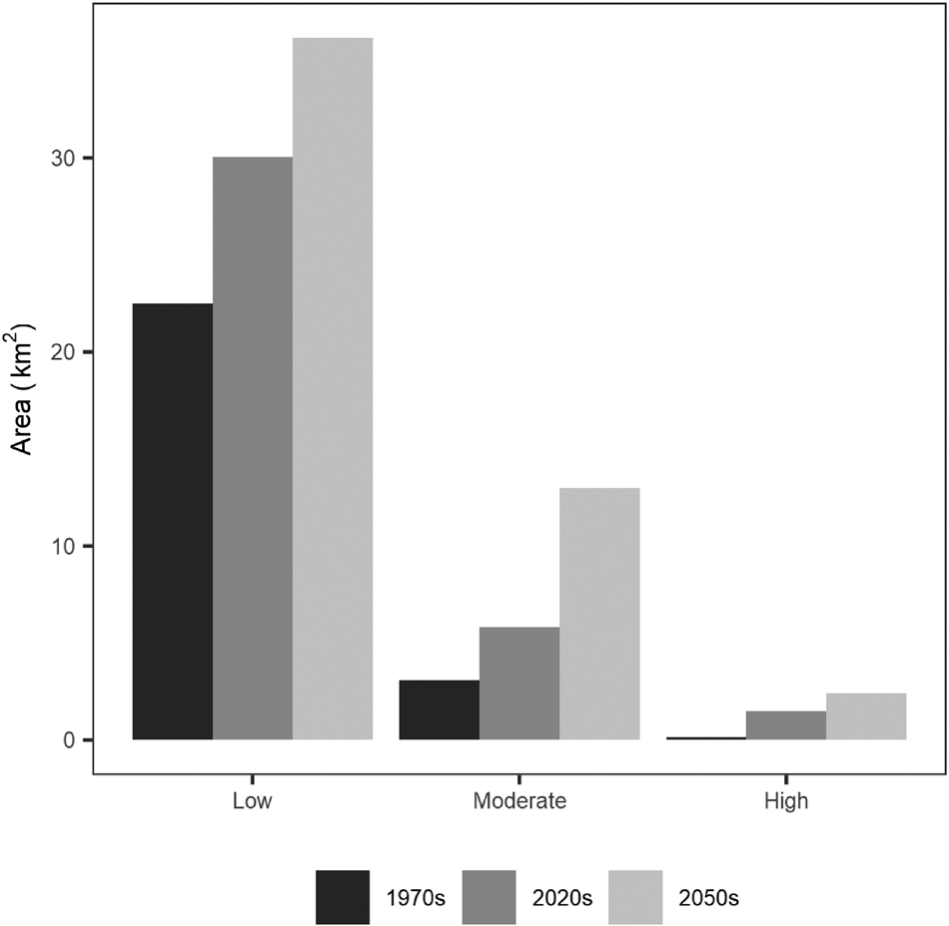
The extent of low (1 to 4), moderate (4 to 9), and high (greater than 9) values of the human-bear conflict risk index as modeled for the 1970s, current, and 2050s time periods. The extent and intensity of human-bear conflict risk increased through time in the simulation. Programs used to create figures: Figs. 1 to 4 were created using ArcMap from rasters generated by ALCES Online; Fig. 5 was created using R

High connectivity and recreational activity in natural land cover surrounding settlements resulted in elevated risk of human-bear conflict to the south of Canmore and to the north of the Banff townsite (Fig. 4). Human-bear conflict risk increased during the simulation, with the spatial extent of moderate- or high-risk areas more than doubling during the historical period and again during the forecast period (Fig. 5). Risk expansion was greatest around Canmore in the historical simulation, a pattern that continued in the Base Case forecast wherein risk expanded eastwards in response to the simulated expansion of Canmore and associated recreational activity (Fig. 4).

By limiting recreational activity and development footprint, all three mitigation scenarios substantially reduced human-bear conflict risk compared to the Base Case (Fig. 5). The Limited Urban Expansion scenario, which excluded urban expansion and associated growth in recreational activity from the eastern portion of Canmore known in planning documents as the Area To Be Determined, reduced the extent of moderate- or high-conflict risk areas by 35% relative to the Base Case. The No Informal Trails scenario, which eliminated recreational activity from informal trails, had the largest impact, achieving a 41% reduction in moderate- or high- conflict risk areas compared to the Base Case scenario. In contrast, the Restricted Recreation scenario, which reduced recreational activity by 50% across the study area, led to a 23% decline in moderate- or high- conflict risk areas compared to the Base Case scenario. The larger impact of eliminating informal trails compared to reducing overall recreational activity was due to the prevalence of informal trails in proximity to townsites where least cost paths were also abundant.

## Discussion

Integrating landscape simulation and least cost path analysis provided the broad temporal and spatial perspective needed to highlight key issues facing the coexistence of grizzly bears and humans in the Bow Valley. How grizzly bears use the landscape likely has and will continue to be impacted by development footprint because bears and humans both prefer the flat valley bottom. An implication of this overlap is that the impact of development footprint on connectivity is disproportionate to the footprint’s relatively small area. The locations of the townsites of Banff and Canmore were likely corridors for grizzly bear movement prior to settlement, and their development has gradually diverted the corridors upslope. Continued settlement expansion in the valley is likely to further divert movement corridors, here modeled as least cost paths, away from preferred habitat. The highway also alters connectivity by impeding movement across the valley bottom except at crossings, thereby isolating otherwise suitable habitat. Project-based environmental assessments are ill-suited for identifying these issues because their narrow scope is insufficient to capture cumulative effects of multiple developments and to address landscape context.

While important, fragmentation of habitat by development footprint is only part of how humans impact grizzly bears in the region; human-bear interactions are also an important risk factor. Provincial bear management areas that overlap with the study area experience rates of human-caused grizzly bear mortality and translocation that are estimated to exceed the threshold for population stability (Alberta Environment and Parks 2020). In the Bow Valley, recreational activity is prevalent along both designated and informal trails, especially around the periphery of settlements. The number of informal trails for walking, biking, hiking, scrambling, or off-highway vehicle use continue to expand year over year (Farr et al. 2017, Whittington et al. 2022). Whether built for a specific type of recreational experience or worn in over time as connectors between backyards and established trail networks, informal trails are much easier to create than to restore (Johancsik 2016). We used a human-bear conflict risk index to identify areas where natural land cover is likely to have high connectivity value for wildlife and be used for recreation, thereby creating risk of human-bear interaction. The locations of moderate and high values of the human-bear conflict risk index are consistent with the locations of aggressive bear incidents near the Town of Banff including Tunnel Mountain, Mount Norquay, and towards Lake Minnewanka (Bow Valley Human-Wildlife Coexistence Roundtable 2018) and locations of human-grizzly bear conflict and grizzly bear sightings near Canmore such as the Nordic Centre and the Three Sisters area (Ellis 2022; Hudes 2019; Rocky Mountain Outlook 2016).

The projected increase in human-bear conflict risk under the Base Case scenario is not a prediction, but rather illustrates a threat associated with the present management paradigm in the region. Irreducible uncertainty and contingency are such that simulations will never accurately predict the future. Rather, the benefit of cumulative effects scenario modeling is to compare the consequences of a range of scenarios (Peterson et al. 2003). Especially when done in collaboration with management agencies and stakeholders, scenario modeling can build a shared understanding of strategies that are consistent with desired outcomes (Thekdi and Lambert 2014). This project examined three types of mitigation strategies identified through discussion with the advisory group: limiting development within and close to high connectivity areas (Limited Urban Expansion scenario); limiting recreational activity to a fixed set of designated trails (No Informal Trails scenario); and limiting the overall amount of trail use (Restricted Recreation scenario). Simulating coarse implementations of these strategies were not intended to be realistic but to provide an initial assessment of potential benefits. Outcomes suggest that limits to development and recreational activity both have the potential to substantially mitigate growing human-bear conflict risk in the region. This outcome, while logical in hindsight, was enlightening given that recreational activity was not a focus at the outset of the analysis. Project-based impact assessments generally do not provide an opportunity for key environmental outcomes to be discussed among multiple jurisdictions and prioritized, nor for consideration of the suite of strategies by which to achieve these outcomes. Bringing together the jurisdictions of the Town of Banff, Town of Canmore, and Alberta Parks was one of the strengths of our cumulative effects modeling. It is important to note, however, that other jurisdictions including Parks Canada, the MD of Bighorn and Stoney Nakoda First Nations chose not to be involved, which likely limits the relevance of this work to those groups.

It is particularly important to note that our modeling advisory group did not include local First Nations. The Stoney Nakoda First Nations declined to participate, which may be due to any number of factors including consultation fatigue, limited capacity, or requirements to engage with Crown referrals on industrial projects (e.g., see Persaud et al. 2020). Neither this modeling project nor municipal development projects in Alberta have a Crown-referred duty to consult; nor do they offer financial supports for First Nations involvement; nor do they confer decision-making authority on First Nations on land-use decisions that impact the practice of Treaty Rights. The role of Indigenous Peoples as sovereign decision-makers in land use decisions is a structural problem in Canada, and one that is far from resolution. Locally, this is evident in the Stoney Nakoda First Nations’ involvement in public hearings and legal proceedings regarding Canmore-based development plans, on the basis of inadequate (but not clearly legally required) consultation (e.g., Colgan 2021, 2022). Legal questions remain about the application of duty to consult and honor of the Crown at different levels of government and different land use applications, and these questions loom over but are beyond the scope of this paper.

Other limitations of the work are rooted in assumptions made in the absence of more complete or accurate data. Outcomes regarding the risk of human-bear conflict are contingent on assumptions regarding the activity patterns of humans and grizzly bears. The use of least cost path analysis to infer areas more likely to be used by grizzly bears assumes that bears maximize habitat and minimize distance as they move between locations. Grizzly bear behavior is also influenced by other factors such as availability of food, and this study is not designed to assess important sources of risk such as attractants in townsites and vehicle collisions. Constraining least cost paths to the study area likely also exaggerates the importance of east-west movement. In terms of modeling human activity, Strava data provide an incomplete representation of recreation and likely under-represent activities whose practitioners are less likely to use the app, such as dog walkers. As well, because Strava data were only available for the current time period, temporal changes in recreational activity were assumed to be linked to changes in settlement footprint and human population. Other patterns are possible, such as more rapid growth in activity if recreation is driven by visitors, or slower growth if residents of new developments recreate less than anticipated in the surrounding landscape, or if recreation restrictions are actively enforced in wildlife corridors. Further, the approach of modeling change in recreational activity and trails by extrapolating current spatial patterns is simplistic and could be improved upon by adopting approaches that were beyond the scope of this study, such as agent-based modeling to simulate human recreational behavior (e.g., Itami et al. 2003), statistical modeling of the relationship between recreational activity and environmental characteristics (e.g., Sun et al. 2017), and simulation of trail formation (e.g., Helbing et al. 1997). Despite these limitations, the spatial pattern of modeled human–bear conflict risk was consistent with observations from the Bow Valley (Bow Valley Human-Wildlife Coexistence Roundtable 2018; Hudes 2019; Ellis 2022), and other studies have also identified impacts of human activity on wildlife in the research area (Gibeau 1998; Gibeau et al. 2002; Rogala et al. 2011; Whittington et al. 2022). Given the potential impact of recreational activity to wildlife in natural landscapes, more monitoring and research is needed to better understand the scope of recreational activity, its change through time, and its effects.

Land managers are simultaneously empowered and constrained by the specific legal and social contexts in which they operate, including but not limited to strategic plans, policy, and legislation, economic benefits, community values, and private land owner rights. In the Bow Valley, there is high social license for prioritizing the environment and coexistence with wildlife, but few avenues for the regional perspective or shared decision-making that a cumulative effects framework suggests is necessary. The Bow Valley is considered a leader in human-wildlife coexistence among mountain communities (Bow Valley Human-Wildlife Coexistence Roundtable 2018). The Towns of Canmore and Banff are particularly progressive on this front, with robust wildlife education programs, early adoption of bear-proof garbage and composting bins, and comprehensive bylaws to manage artificial and natural wildlife attractants. These communities are also unusually wealthy and educated for municipalities of their size, factors that likely contribute to high levels of informed civic engagement (e.g., CBC News 2021). Consequently, Bow Valley communities are better positioned to prioritize wildlife conservation in their decision-making than many other locations.

Despite these advantages, a challenge facing planning for human coexistence is that Bow Valley jurisdictions have notably diverse decision-making contexts. Because the Town of Banff is situated within a National Park, its development footprint is fixed and decisions for trails and infrastructure around the town must adhere to Parks Canada’s policies to ensure ecological integrity. Land-use decisions within the Town of Canmore and the MD of Bighorn, however, are governed by the provincial Municipal Government Act as well as local Municipal Development Plans (MDPs), none of which prioritize the environment. Municipalities are required by the province of Alberta to have MDPs primarily to identify and plan for development opportunities and economic growth (Alberta Municipal Affairs 2015). Development decisions are necessarily made locally, and even when proposed development footprints extend across jurisdictional boundaries, the project components within each jurisdiction are generally considered separately by distinct decision-makers. Fragmented decision-making is a challenge when it comes to grizzly bear management and population recovery. Human-bear interaction is highlighted as a threat in the most pertinent policy tool for grizzly bear management, the provincial Grizzly Bear Recovery Plan (Alberta Environment and Parks 2020). The recovery plan states that recovering grizzly bear populations in major transportation corridors like the Bow Valley requires “working with the responsible provincial and municipal government agencies to ensure that grizzly bear movement needs are considered in development decisions” (Alberta Environment and Parks 2020, p. 59). However, no mechanism exists in Alberta to ensure that different levels of government work together to make these decisions—nor for land-use decision-making processes to include Indigenous Peoples’ perspectives.

To ensure that environmental assessment processes effectively evaluate impacts beyond the local and short-term geographic and temporal scales, cumulative effects analyses should be incorporated and evaluated on the scale at which the key ecological attributes operate. Identifying threats and mitigations for wide-ranging species almost certainly requires broadening the geographic scale beyond that of any single jurisdiction, especially in regard to municipalities. When the appropriate scale for analysis exceeds one jurisdiction’s boundary, the assessment process should require the involvement of multiple land managers for different jurisdictions in the region, something that is not currently required or typically considered. It is clear that the existing project-based EA process is ill-suited to this task, though there is no obvious alternative point of intervention, either. Consequently, while is some value in cumulative effects scenario modeling at the project scale, it may be a more promising tool for land use planning, land use and development policy, and related processes at the regional scale. Recognition of the value of scale-appropriate planning and evaluation to assess the cumulative effects of human and resource use in decision-making is not new (e.g., Hunsaker and Williamson 1992, Hunsaker 1998) but the adoption of comprehensive and effective processes have largely been slow and complicated (Harriman and Noble 2008, Gunn and Noble 2011, Council of Canadian Academies 2019).

Wherever land use planning occurs, we recommend that land managers jointly determine the desired state for the ecological region or species in question, such as retaining or improving grizzly bear connectivity, or ensuring that human-wildlife conflict risk in the region does not exceed a certain threshold. The involvement of decision-makers who operate at different geographic scales, like municipal land managers, provincial biologists, and park managers, for example, also encourages sharing of data, plans, strategies, and policies among jurisdictions, and ultimately, encourages collaboration toward common goals. Cumulative effects scenario modeling should not and cannot be the responsibility of community groups and non-profit organizations, who as a rule are over-burdened and under-resourced. Such a practice should be part of good land use planning beyond project-based EA, especially in areas where human activity and at-risk species are known to be a challenge, as is the case in the Bow Valley.

We also recommend modeling past, present, and future scenarios. For this project, modeling historic movement connectivity allowed a clearer understanding of the degree of change that has occurred to date; it ensured that the present-day state was not the only baseline from which future change was measured. This helped to show that undeveloped land remaining near towns in the Bow Valley is rare and valuable for grizzly bear movement paths. Scenario modeling allows land managers to consider the potential impact of different management strategies on future outcomes, illustrating that decisions made now can change the course of the future. Scenarios also allow managers to consider the costs and benefits of different conservation strategies. Scenarios are not meant to be prescriptive, but rather a starting point for more nuanced conversations that include community members and Indigenous Nations in the region.

Finally, we recommend transparent and clear communication throughout the EA process with everyone who has a stake or interest in the region. While deep and meaningful community engagement processes were outside the scope of this project, we did ensure that user groups, community members, and government staff were informed about the work being done via a webpage, updated blog posts, final presentations, and discussions with stakeholder groups and government, and wide distribution of a final report and executive summaries. While time-consuming and expensive, open communication ensured a certain level of buy-in in the work rather than fear around pre-determined outcomes. Only through strong communication and collaboration can environmental assessment, or land use planning more generally, achieve the scope required to avoid continued erosion of connectivity for grizzly bears and growth in human-bear conflict.
